# From seeing to showing: towards a neuroscience of emotion production

**DOI:** 10.1098/rstb.2025.0136

**Published:** 2026-09-24

**Authors:** Seohyeon Lee, Solha Hwang, M. Justin Kim

**Affiliations:** ^1^ Department of Psychology, Sungkyunkwan University, Seoul 03063, Republic of Korea; ^2^ Department of Brain Science and Engineering, Sungkyunkwan University, Suwon 16419, Republic of Korea; ^3^ Center for Neuroscience Imaging Research, Institute for Basic Science, Suwon 16419, Republic of Korea

**Keywords:** emotion expression, facial expression, production of facial expressions, emotion experience, affective neuroscience, fMRI

## Abstract

Facial expressions are considered a critical feature in affective neuroscience. Consequently, extensive research has focused on how emotional facial expressions are perceived, including investigations into which brain regions are activated during their observation and how the brain processes their social significance. To advance a more comprehensive understanding of emotion, it is essential to examine all three aspects—perception, experience and production—in a balanced manner. From an evolutionary perspective, the production of facial expressions during emotional experiences is particularly significant, as it reflects both adaptive communication and survival functions. Nevertheless, a review of existing databases reveals that research on the production of facial expressions remains scarce compared with studies on perception. This gap may stem from several methodological constraints, including the difficulty of reliably inducing emotional experiences in laboratory settings, the challenge of recording participants’ full facial expressions in a magnetic resonance imaging scanner and limitations in current analytical approaches for facial expressions. In this review, we argue for the importance of studying the production of facial expressions and outline potential strategies to address these challenges.

This article is part of the theme issue ‘Mechanisms, development, phylogeny and functions of emotional expressions’.

## 1. Introduction

Emotions are intimately intertwined with human life, and despite their longstanding appeal to emotion researchers, there remains considerable disagreement regarding how they should be defined. Nevertheless, most researchers agree that emotions serve important functional roles. Emotions are usefully characterized as functional states that are elicited by certain events (i.e. stimuli or antecedent processes) and that, in turn, generate downstream events such as behaviours [1]. A unique aspect of emotion is that many different antecedent processes can cause the same type of emotional state (i.e. the flexible nature of emotion). These emotional states manifest in observable behavioural changes, such as facial expressions and vocal tone, as well as in less directly observable biological and physiological responses (i.e. somatic responses), including changes in heart rate, respiration and autonomic activity. Furthermore, social contexts shape how external stimuli are perceived, how we interpret our internal state and how ensuing emotional states are expressed.

Among the many ways humans express emotions, facial expression is among the most representative channels. Facial expressions—visible patterns of muscle activity in the face—are among the most fundamental forms of human expressions, serving as a primary means of conveying emotions [2]. Facial expressions communicate emotional states in an intuitive and immediate manner and serve as a critical channel for understanding and responding to others’ emotions in social interactions [3]. Such affective information is rarely confined to a single channel; facial expressions simultaneously transmit multiplexed signals that encompass dimensional (e.g. valence and arousal) as well as categorical (e.g. happy and fearful) aspects of emotions, which can be effortlessly decoded by human observers [4].

In parallel with their significance in everyday social life, facial expressions have long been regarded as a central focus in the scientific study of emotion [5]. Darwin first proposed that emotional expressions have evolved as adaptive features, with facial expressions of emotion representing universal and innate products of this adaptive process [6]. Ekman conducted cross-cultural research and provided support for Darwin's claim, arguing that basic emotions, such as happiness, sadness and anger, are expressed through specific corresponding facial expressions that can be recognized universally [7]. Since then, facial expressions of emotions have been widely studied and used in the field of affective science [8].

However, most laboratory-based affective science research—especially research taking a cognitive neuroscience approach—heavily focuses on the perception rather than the experience of emotions conveyed by facial expressions [9]. At present, research on the neural basis of facial expression production remains limited. This gap may reflect both an extensive history of face perception research in cognitive psychology and neuroscience [10,11] and methodological challenges in neuroscience, such as the artificial constraints of functional magnetic resonance imaging (fMRI) environments, the high costs of MRI-compatible recording equipment and the technical difficulty of accurately capturing facial movements during scanning—all of which warrant improvement. We believe that elucidating the mechanisms underlying emotion expressions by studying the production of facial expressions may contribute to the advancement of emotion research. How people communicate their experienced emotions through facial expressions is not the same as how they perceive facial expressions [9]. A deeper insight into the mechanisms of emotion also requires focused investigation of how facial expressions are generated. Understanding when, how and why facial expressions of emotion are displayed requires a broader investigative scope, spanning behavioural to neural levels, within the context of emotional experience and production.

Accordingly, we will briefly revisit key findings from research on the perception of facial expressions. Then, we will outline why an explicit focus on the neural mechanisms underlying the production of facial expression has become increasingly necessary. Next, drawing on a review of prior studies, we will describe current trends in production research and, finally, discuss what methodological considerations are essential for studying them effectively in neuroscience.

## 2. Perception of facial expressions

The history of facial expression research can be usefully discussed in the context of Darwin's theory of evolution by natural selection [6]. Darwin proposed the concept of adaptation, which implies that organisms with traits that are advantageous for survival and reproduction in a particular environment are more likely to pass those traits onto their offspring. According to Darwin [6], facial expression was one of such traits, highlighting an adaptive function to humans—social communication. Following this line of thought, Paul Ekman, from his cross-cultural research, argued that there are universally recognizable facial expressions of emotions [7] and suggested that those emotions should be called basic emotions [12]. The basic emotions that putatively exist in all cultures include happiness, sadness, anger, fear, disgust and surprise, and more complex emotions can be made through their combinations [12–14]. The early dominance of the basic emotion theory (BET) in emotion research for decades may have contributed to a focus on the perception aspect of emotional facial expressions [12,15–20]. Beyond that, several factors may have contributed to the field's greater emphasis on perception-oriented studies employing facial expressions. First, many studies intentionally and appropriately used facial expressions because of their functional characteristics. Traditionally, their main function has been considered as social communication [21]. Elucidating how we perceive and interpret others’ facial expressions, along with the underlying neural mechanisms, is crucial for understanding socioaffective functioning. Second, given the early influence of BET, facial expressions of basic emotions are considered to be perceived in the same manner, regardless of different cultural backgrounds. This assumption aligns with the broader goal of psychological research—to generate conclusions that extend beyond the specific sample studied and apply to humans more generally. Unlike language, which is inherently bound by culture, facial expressions of emotion, therefore, were an attractive choice as experimental stimuli in emotion research. Third, over the past multiple decades, numerous validated facial expression stimulus sets have been developed and made widely accessible for research (e.g. [22–28]). Such standardized and well-validated datasets have been crucial for experimental control—a central requirement in psychological science—because they allow researchers to systematically regulate features such as expression category, identity, race, pose, luminance and image quality. Moreover, from an ethological perspective, human facial expressions serve as reliable stimuli capable of eliciting consistent responses among conspecifics [29,30], exhibiting stability and utility in experimental settings. As a result, using photographs or videos of posed expressions has become common practice in neuroscientific research.

For these reasons, most studies involving facial expressions might prefer perception over production [31]. Nevertheless, we have had significant insights into the mechanisms of emotional processing from the studies about the perception of facial expressions. Studies involving young infants revealed that the perception of facial expressions might be an inherent skill that starts to develop from early life; empirical evidence suggests the existence of the ability to perceive facial expressions and discriminate between emotions at a very early stage of development [32–34]. The finding that infants can differentiate facial expressions suggests that face perception constitutes a foundational mechanism for the emergence of social interaction skills. This early sensitivity to emotional cues not only facilitates bonding and communication with carers but also sets the stage for the development of more complex socio-emotional competencies throughout childhood. In addition, it is worth noting that, in cognitive neuroscience, substantial advances in understanding the neural mechanisms of face processing have largely stemmed from research focused on perception. Kanwisher and colleagues have famously coined the term ‘fusiform face area (FFA)’ [10] and have proposed that this region is a module that is specialized for face perception [35–37]. Evidence suggests that the FFA is involved in judging emotional expressions as well, showing that greater FFA activation is associated with facial expression even when attention is directed towards identity, and that emotional faces elicit stronger FFA activation than neutral faces during passive viewing [38]. Other regions involved in perceiving facial expressions include the amygdala [39,40] and the superior temporal sulcus (STS) [41,42]. The amygdala is known as an essential link in the response to stimuli with emotional processing [43]. Consistent with this function, damage to this region impairs the processing of fearful facial expressions [44]. This finding underscores the amygdala's crucial role in the detection and processing of fear/threat signals. Additionally, the STS is a critical region within the social brain network [45]. In particular, the STS constitutes a core component of the face-processing system and is specialized for detecting changeable aspects of faces [11]. Neuroimaging studies have consistently reported atypical activity in this region during face processing in individuals with autism spectrum disorder [46], a neurodevelopmental disorder characterized by core social interaction deficits [47]. These findings suggest that impairments in face processing are closely associated with disruptions in social functioning.

## 3. Production of facial expressions

Facial expressions represent more than reflexive reactions to external stimuli; they integrate the internalization of such stimuli into subjective emotional experiences and their subsequent externalization through expressive behaviour. Accordingly, research on facial expressions encompasses three core domains of emotion: perception, experience and production. Perception, as discussed above, is the most extensively investigated domain, which entails the detection of facial cues and the extraction of their emotional significance. Experience refers to the integration of perceptual input with one's internal states, yielding subjective affect that transcends simple stimulus–response associations. Production denotes the expression of such affect through facial musculature. A comprehensive account of facial expression, therefore, requires that these processes be examined in an integrated framework. Previous studies have already attempted to address this integrative perspective. A representative example is the classic work by Landis [48], who elicited spontaneous facial expressions using a wide range of stimuli (e.g. music, odours and even electric shocks) and compared them with expressions deliberately posed in the absence of emotional experience. This study reported substantial individual variability in spontaneous expressions during emotional episodes and noted that posed expressions—despite lacking genuine concurrent emotional experience—more closely resembled what were considered typical facial expressions. Other studies have combined facial expression perception and production to gain insights into the link between emotional experience and expression. For instance, Marsh and colleagues demonstrated that observers were more accurate in inferring a target's nationality from emotional facial expressions than from neutral expressions, suggesting that cultural or national identity can be implicitly embedded in emotional expressions [49]. Similarly, Elfenbein and colleagues examined facial expression perception and production jointly and identified cultural differences that emerged across both processes [50]. These findings highlight that facial expression cannot be fully understood without considering how culturally shaped expressive patterns are perceived. At the same time, within the ethological tradition, perception has long been treated as a methodological tool for interpreting behavioural expressions as functionally meaningful signals and for understanding their role in communication [51,52]. This perspective suggests that perception-based approaches have long served as methodological tools for studying facial expression production, and that perception studies have provided valuable insights into the nature of emotional facial expressions. Building on these findings, research on facial expression production can be conducted in a more systematic and theoretically grounded manner. Together, these lines of research suggest substantial individual and cultural variability in emotional experience and emotion perception, increasingly highlighting that emotion is more variable than originally proposed by Ekman [53–56]. In this sense, joint consideration of perception, experience and production allows for a more comprehensive and nuanced understanding of emotion.

According to previous reviews [57], two broad perspectives have been proposed regarding facial expressions: the communicatory function and the emotional experience function. The communicatory perspective traditionally regards facial expressions as central to social interaction, providing rapid and efficient signals of an individual's internal affective states [58–60]. By contrast, the emotional experience perspective posits that emotional expressions are automatic displays that arise directly from the individual's emotional experience [6,61–63]. Such perspectives emphasize the role of innate neural mechanisms in shaping facial expressions, particularly for highly salient emotions. However, whether facial expressions reliably mirror underlying emotions remains highly contested [64–68]. These debates challenge the assumption that facial expressions are straightforward read-outs of internal emotion. In line with this critique, the behavioural ecology view conceptualizes facial expressions as products of both emotional and social factors, emphasizing strategic modulation rather than automatic emotional output. Accordingly, internal emotional–motivational processes and overt facial expressions may be partially dissociable, particularly in social contexts where display rules operate. Display rules are learned norms that reflect socially shared values regarding the appropriateness of emotional behaviour and they guide how emotional expressions are managed, depending on social circumstances [69]. For example, individuals in collectivistic cultures may exhibit more similar facial responses to the same antecedent events compared with those in individualistic cultures, whereas more conservative cultural contexts may encourage greater restraint in overt facial expression.

Beyond spontaneous expression, facial expression production is also strongly influenced by emotion regulation processes, particularly expressive suppression. Suppression involves the intentional inhibition of expressive behaviour after emotion generation and is associated with reduced outward expression, minimal changes in subjective experience and increased physiological arousal [70]. Consistent with this idea, converging evidence from neuroimaging studies indicates that suppression engages a distributed network of brain regions involved in cognitive control and motor inhibition, including prefrontal areas and subcortical structures [71–75].

Taken together, clarifying how facial expressions relate to emotional states—and what they actually convey—may be better achieved through studies of facial expression production rather than perception alone. Because the translation from internal emotion to overt facial behaviour involves multiple intermediate regulatory processes, approaches relying solely on observable behaviour or self-report may not fully capture the underlying mechanisms. Neurophysiological measures can, therefore, provide a crucial window into internal emotional and regulatory mechanisms that are otherwise inaccessible through external observation.

Under typical conditions, internal experiences are expressed through a sequential neural cascade linking limbic circuits, frontal–striatal–motor regions and facial effector systems [76]. Disruptions or weakened connectivity within this pathway may result in deficits of expression production, and clinical populations with impaired facial expression offer important clues for inferring the underlying mechanisms. For instance, Möbius syndrome patients retain the capacity for emotional experience but cannot generate expressions owing to congenital underdevelopment of the facial (CN VII) and abducens (CN VI) nerves, resulting in paralysis of the facial musculature [77]. In schizophrenia, by contrast, facial expression is not entirely absent but often appears flattened or incongruent, reflecting affective disturbances [78]. Given that deficits in facial expression production vary across disorders, their fundamental mechanisms cannot be fully understood through behavioural observation alone. Functional neuroimaging approaches, however, extend beyond the documentation of overt expressive behaviour to capture the implicit transformation from experience to expression, thereby offering a useful methodological framework. Altogether, these points highlight that, despite facial expressions comprising perceptual, experiential and expressive components, the production component remains comparatively understudied—particularly within neuroscience. This gap is not merely methodological but conceptual—without understanding how subjective emotional experience is transformed into observable expressive behaviour, our account of emotion remains incomplete. Consequently, advancing neuroscientific research on expression production is essential for building an integrated model of emotion that spans experience, regulation and motor implementation. Although perception-focused research has undeniably led to major progress in the study of emotion, we believe that more extensive research on facial expression production under emotional experience in neuroscience holds great potential for driving the next wave of advances in emotion science.

As noted earlier, emotion can be conceptualized as a functional process in which external stimuli evoke internal states that manifest in both observable and unobservable responses [1]. The lack of consensus on a universal definition of emotion may reflect the field's limited integration of psychological, behavioural and neural perspectives across this full sequence of processes. For instance, if emotions evolved primarily for social communication, why do individuals display emotional facial expressions even in the absence of observers? This possibility implies that emotions may serve additional biological functions beyond signalling to others. Understanding the neural mechanisms that support facial expression production during emotional experience can, therefore, help build a more integrated account of emotion.

Three points illustrate this potential. First, neuroscientific approaches can clarify how subjective emotional experience is linked to expressive behaviour. Because emotional states in humans inherently involve subjective experience, understanding how such experience unfolds and drives expression is essential. Neural measurements allow researchers to examine the emergence and temporal dynamics of emotional experience, regulation and expression—phenomena that are often difficult to disentangle using self-report or behavioural observation alone. High-temporal resolution tools, in particular, make it possible to trace whether regulation occurs after the onset of experience, whether it is engaged early in the experience or whether it acts specifically on the expressive stage.

Second, neural data can help differentiate expressions arising from genuine emotional experience from those shaped by social or regulatory demands. Whereas activation in emotion-related circuits may reflect spontaneous affective responding, recruitment of prefrontal regulatory networks may indicate socially modulated or deliberately controlled expressions. Thus, two expressions that appear behaviourally similar may be driven by distinct neural processes. Indeed, Wild and colleagues confirmed separate cortical regions responsible for the production of emotionally driven spontaneous smiles versus voluntary smiles [79]. This approach also enables researchers to investigate how social context influences the coupling between emotional experience and expression.

Third, examining the neural basis of expression production can help identify which aspects of emotion are universal and which are culturally or individually variable. If particular emotional experiences consistently elicit stable neural signatures and emotion-specific facial motor patterns across contexts, such evidence would support a basic emotion framework. Conversely, if neural activity during expression production varies systematically with context, goals or conceptual processing—even when self-reported emotion is similar—this would align with the constructionist account of emotion. Parsing the sources of variation in emotional experience and in expression generation is, therefore, crucial for differentiating between these theoretical perspectives.

Taken together, neuroscience provides complementary tools to dissect the relationship between emotional experience and facial expression production at temporal, mechanistic and circuit levels. For example, instructing participants not to display a fear expression during exposure to a fear-evoking stimulus would allow researchers to examine how amygdala activity changes under intentional suppression, thereby providing additional evidence regarding how neural patterns relate to emotional experience, regulatory processes or the motor implementation of expression. When considered alongside behavioural and physiological measures, such neuroscientific approaches can contribute to a more refined understanding of the relationship between emotional experience and expression production, and ultimately a more comprehensive understanding of emotion. Furthermore, facial expressions themselves may serve as an objective complement or alternative to subjective self-reports of emotional states.

Therefore, we will provide a systematic review of the existing research on the production of facial expressions to date and discuss the obstacles hindering active research as well as possible solutions, focusing on the field of cognitive and affective neuroscience.

### (a) Literature search and review

To identify the current trend of facial expression production research in neuroscience, in September 2025, we conducted a literature search in electronic databases using the following keyword combinations through PubMed and PsycINFO: (i) ‘facial expression*’ OR ‘facial movement*’ AND (ii) ‘emotion*’ OR ‘affect*’ AND (iii) ‘produc*’ OR ‘display*’ OR ‘generat*’ OR ‘move*’ OR ‘pose*’ AND (iv) ‘fMRI’ OR ‘functional magnetic resonance imaging’ OR ‘BOLD’ OR ‘Blood Oxygen Level-Dependent’ OR ‘EEG’ OR ‘electroencephalography’ OR ‘neural*’. Search was limited to the title and abstract fields to maximize relevance and reduce retrieval of non-specific records and was not limited by date. Figure 1 presents a flow diagram of the study selection process according to the Preferred Reporting Items for Systematic Reviews and Meta-Analyses (PRISMA) statement [80]. Since each excluded record was assigned a single primary reason for exclusion, when multiple reasons applied, we applied a pre-specified prioritization rule in the following order: (i) not published in English, (ii) not related to the topic of interest, (iii) not a human study, and (iv) review or meta-analysis. During the eligibility assessment, the full texts of all articles were examined. Studies were excluded if the neuroimaging data were not investigated in the context of facial expression production or if it was unrelated to the topic of interest. Ultimately, a total of 49 articles were identified that investigated the production of facial expressions in neuroscience research.

**Figure 1.**
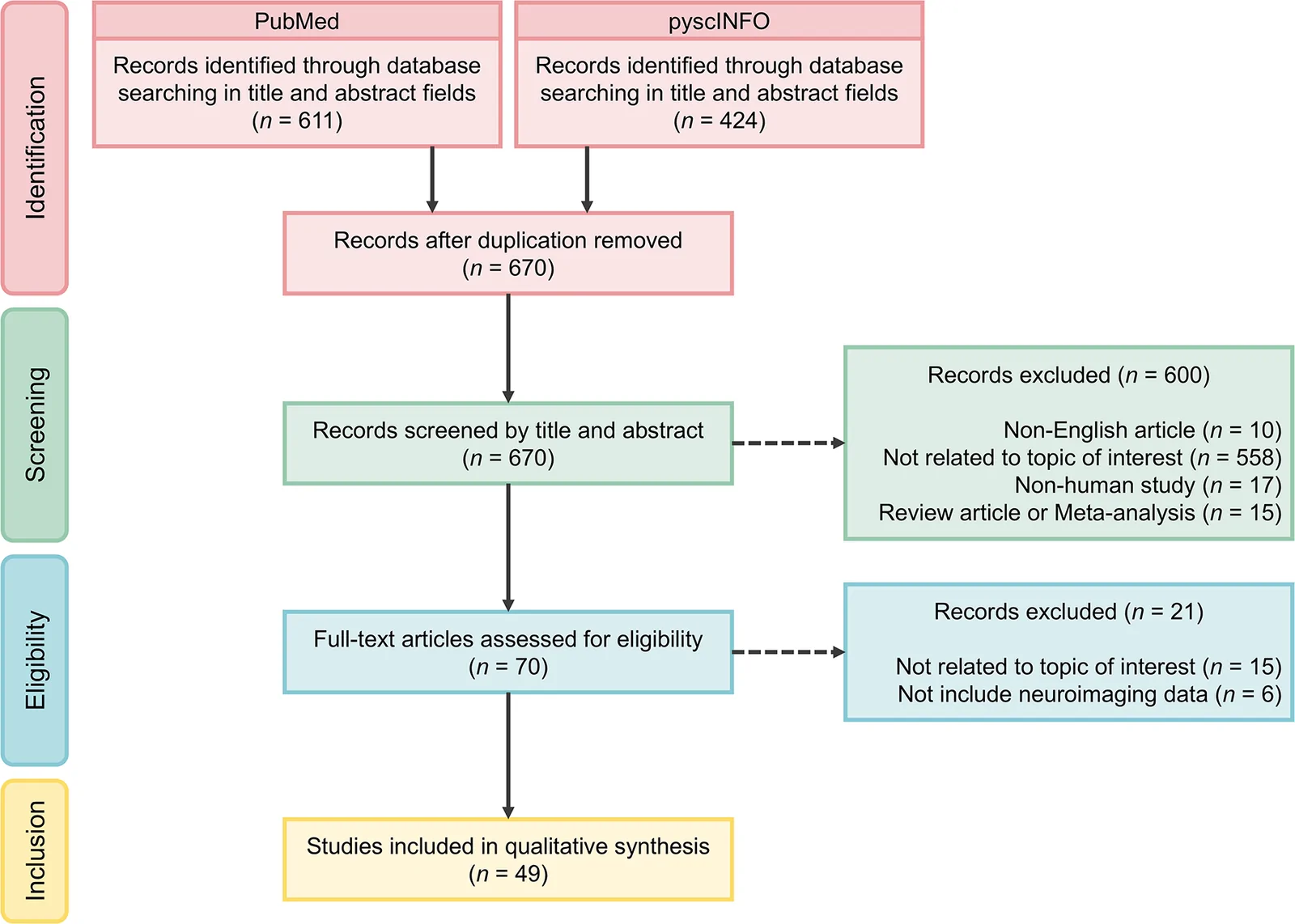
PRISMA flow diagram summarizing the identification, screening, eligibility assessment and inclusion of studies in the literature review.

Furthermore, the 49 included articles were categorized according to the approaches used to evoke facial expressions in participants (figure 2). Four distinct approaches were identified: *experience*, in which facial expressions emerged spontaneously in response to subjective emotional feelings; *mimic*, in which participants were instructed to imitate another person's facial expression; *stimulation*, in which external stimuli were applied to directly induce a facial expression; and *voluntary*, in which participants deliberately produced a specified facial expression without relying on subjective emotional experience. Among these, the experience approach is particularly important, as facial expressions produced through mimic, stimulation or voluntary approaches—although conducted within an affective research context—may rely on mechanisms distinct from those underlying subjective emotional experience. Experience encompasses only spontaneous, naturally occurring facial expressions driven by intrinsic emotions and does not include voluntary expressions produced intentionally. Among the four modalities, only those categorized as ‘experience’ correspond to spontaneous facial expression. We identified only 10 articles that assessed the production of facial expression during emotional experiences (figure 2 and table 1).

**Figure 2.**
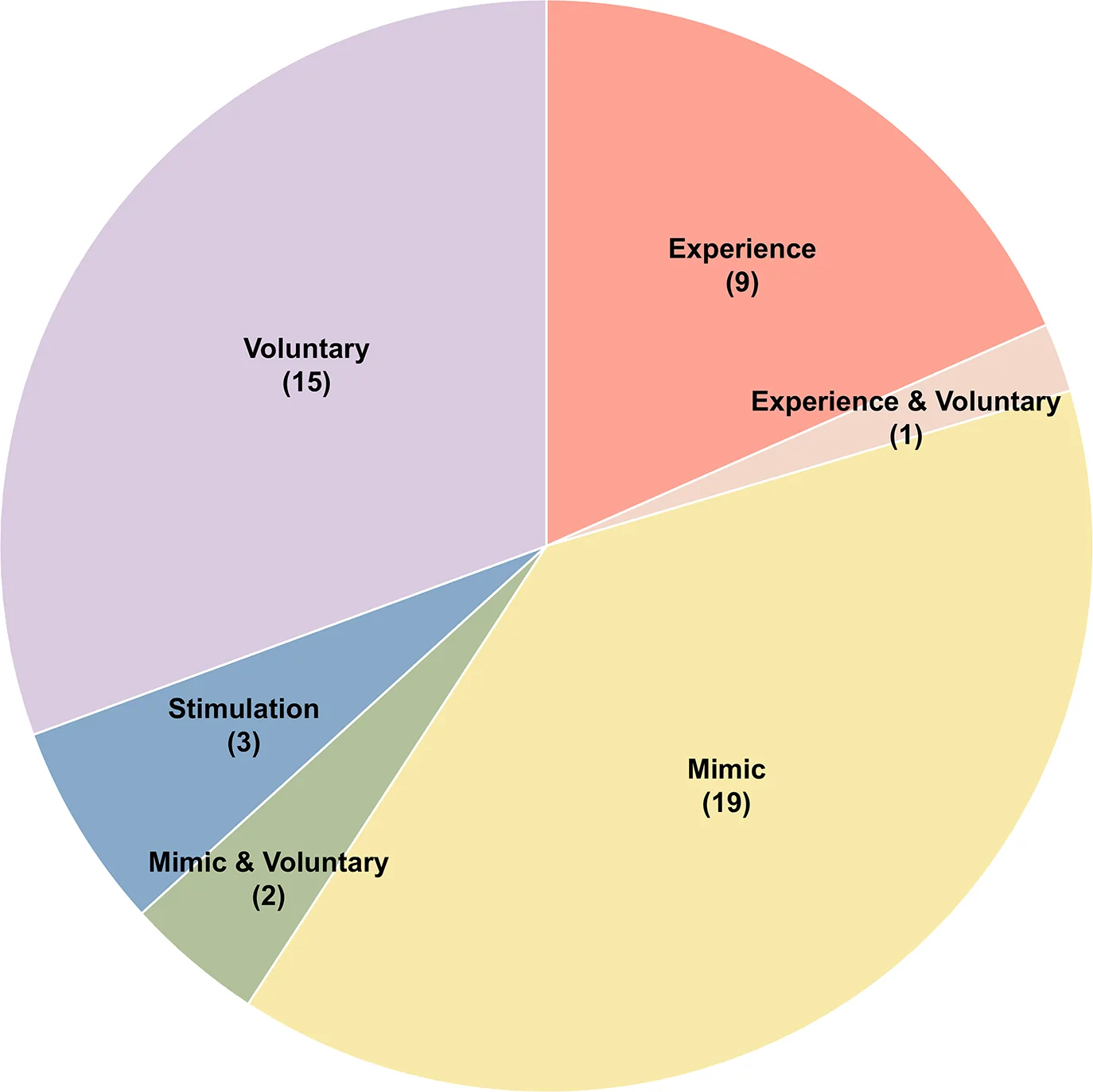
Summary of approaches used to elicit the production of facial expressions in neuroimaging studies (*n* = 49). Numbers in parentheses indicate the number of studies assigned to each category.

**Table 1.** Overview of neuroscience studies on the production of facial expressions elicited through emotional experience. EEG, electroencephalography; EMG, electromyography; FACS, facial action coding system; fNIRS, functional near-infrared spectroscopy.

tudy	emotion elicitation	social context	expression type	expression measure	neural measure
Fox & Davidson [81]	joy and disgust	none (alone)	spontaneous; facial expressions generated with emotional experience	analysed using the maximally discriminative facial coding system (MAX)	EEG
Iwase *et al.* [82]	joy (specifically, laughter)	none (alone)	spontaneous or voluntary	facial EMG; electrodes on bilateral major zygomatic muscles and orbicularis oculi muscles	PET
Goldin *et al.* [73]	disgust	none (alone)	spontaneous (including reappraisal and suppression)	manual FACS coding by human	fMRI
Kunz *et al.* 2011 [83]	pain	none (alone)	spontaneous	manual FACS coding by human	fMRI
Ravaja *et al.* [84]	angry, happy, sad and pleasantly relaxed	social interaction; cooperate with or defect from a virtual character	spontaneous	facial EMG; electrodes on the left corrugator supercilii, zygomaticus major and orbicularis oculi muscle regions	EEG
Grabell *et al.* [85]	frustration	none (alone)	spontaneous	manual FACS coding by human	fNIRS
Zarei *et al.* [86]	joy (specifically, laughter)	none (alone)	spontaneous	automated FACS coding using face reader software	quantitative EEG (qEEG)
Zhao *et al.* [87]	amusement and fear	none (alone)	spontaneous but trying not to show facial expression	manual FACS coding by human	EEG
Kunz *et al.* [88]	pain	none (alone)	spontaneous	manual FACS coding by human	fMRI
Mosley *et al.* [89]	positive affect	none (alone)	spontaneous	automated FACS coding using *OpenFace* [90]	fMRI

*Note*. Studies are listed by year of publication. More detailed information is presented in the supplementary materials.

During the literature search, we noticed that many articles that were retrieved through the search query are related to the perception of facial expression, not to the production of facial expression. More than half of the articles excluded during the screening stage for not being related to the topic of interest were studies related to the perception of facial expression. Therefore, to provide an overview of research trends on emotional facial expressions in neuroscience, we further classified articles retrieved with the same search query according to their topic into two categories: perception and production of facial expressions. These articles were selected according to the following four criteria: (i) written in English, (ii) human studies, (iii) related to the topic of emotional facial expression, and (iv) not review or meta-analysis articles. As shown in figure 3, most of the articles that exclusively examined the perception of facial expressions among a total of 361 articles were classified.

**Figure 3.**
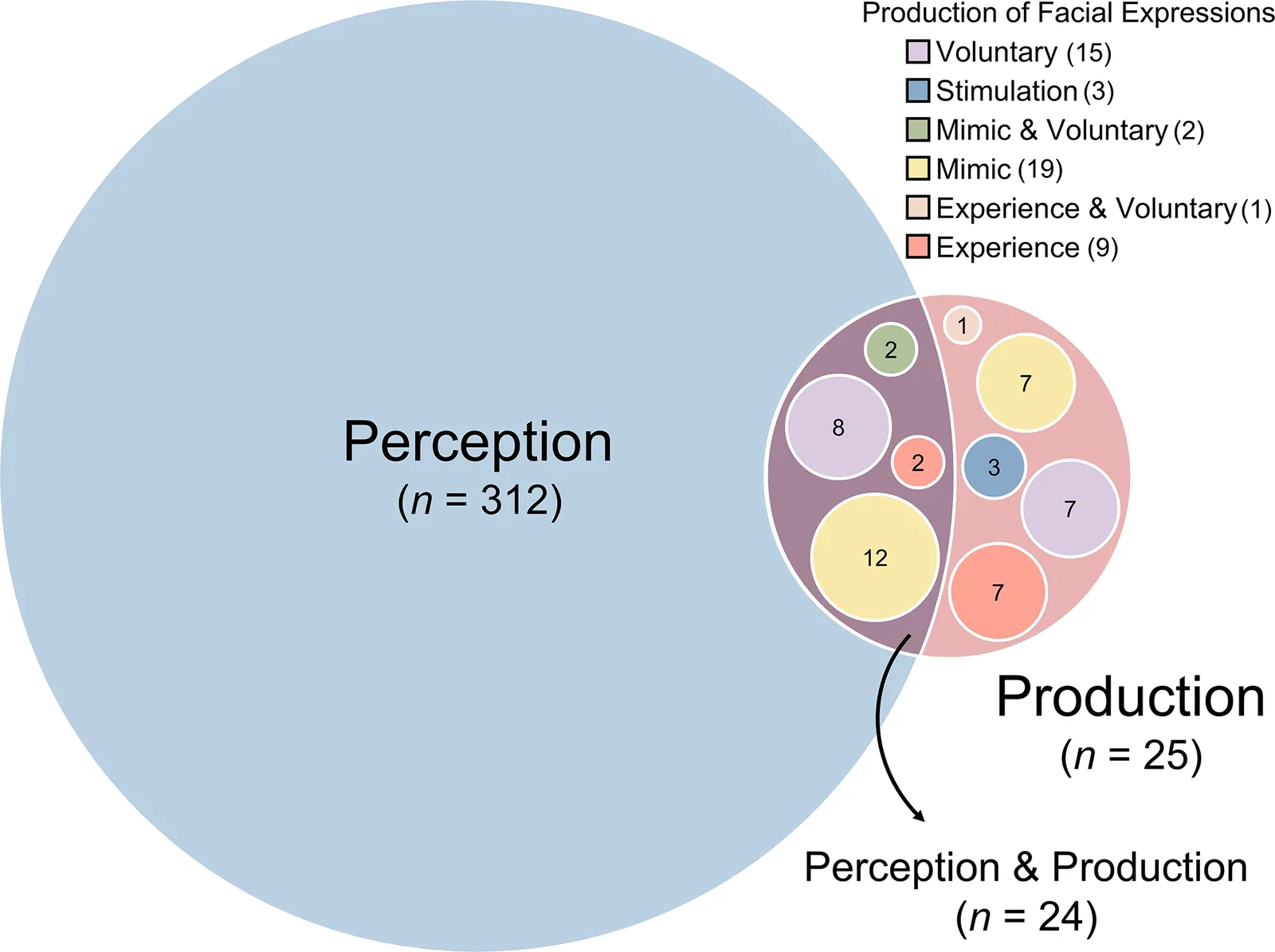
Venn diagram illustrating the categorization of the screened articles (*n* = 361) according to their primary topic into two domains (perception and production) of affective neuroscience research.

### (b) Current state of the field

To date, only a small number of neuroscience studies have directly examined the production of facial expressions during emotional experiences. An overview of the included studies is presented in table 1; detailed methodological information and main findings are provided in the supplementary materials. Although the number of available studies is limited, examining their findings points to several notable tendencies. First, facial expressions do not always map directly onto internal emotional states. In some cases, individuals may display facial expressions that diverge from, or even contradict, their subjective emotional experiences for social reasons [84,85]. It suggests that emotional experience and expressive output may be partially dissociable. Second, even in the absence of explicit awareness, the process through which emotional experiences are externalized as facial expressions appears to involve regulatory mechanisms associated with prefrontal control [83–88]. Third, facial expressions elicited by the same stimulus vary substantially both within and across individuals, highlighting pronounced intra- and inter-individual variability in expressive behaviour. Importantly, several studies further suggest that facial expression production is not a unitary process but can be realized through distinct modes—such as spontaneous under subjective experience versus voluntary expression, different regulation strategies or micro- versus macro-expressions—each engaging partially distinct neural systems. Finally, some studies showed that facial expression production may be a particularly informative window into affective and mood-related disorders [86,89]. Studying facial expressions in conjunction with neural activity holds promise for extending this line of research into clinical contexts. Together, these findings underscore the considerable potential of facial expression production research to provide unique insights into emotional processes spanning from internal emotional experience to overt expressive behaviour.

Despite this potential, the review of the relevant literature revealed a notable gap in affective neuroscience, with only a limited number of studies having examined the production of facial expressions elicited by emotional experiences. There could be several reasons for this. As mentioned above, it is challenging to induce natural facial expressions in experimental situations [31]. Facial expressions are most naturally produced in the context of genuine emotional experiences, such as chatting with friends, watching television or movies or even experiencing pain. These expressions serve not only communicative functions but can also occur spontaneously in the absence of an audience—for example, when we are alone. However, inducing and observing natural emotional experiences pose challenges in neuroimaging research, especially in fMRI studies. To minimize motion-related artefacts during scanning, participants are required to restrict bodily and head movements, a constraint that may severely limit the natural expression of emotion.

A further methodological challenge unique to fMRI research arises from the use of a head coil, which obscures critical regions of the face and thereby complicates the accurate observations of facial expressions. The most widely used approach for analysing facial expression is the facial action coding system (FACS) [91], which characterizes expressions in terms of discrete action units (AUs) based on the anatomy of facial movements. Traditionally, manual coding was conducted by certified FACS coders, a process that is highly time-consuming, subject to coders’ biases and impractical for large-scale video analysis that includes thousands of frames. Recent advances in automated tools, such as *Py-Feat* [92] and *OpenFace* [90], have greatly facilitated the rapid and efficient analysis of facial expressions from photos and videos, thereby reducing many of the limitations of manual coding. Beyond these approaches, recent computer vision methods based on 4D facial mesh models offer a more fine-grained analysis of facial expressions by integrating three-dimensional facial geometry with temporal dynamics. These models enable detailed, frame-by-frame tracking of facial movements and accurate AU prediction [93]. However, those automated methods rely on complete visibility of the face. When portions of the face are obscured—as is often the case in fMRI settings—their accuracy is compromised. For reliable analysis, it is essential to capture the participant's full facial expression. Yet, during fMRI scanning, portions of the face are often hidden or distorted by the coil (see detailed recording outputs in [88,94]). This limitation poses significant constraints on the precise recording and subsequent analysis of facial expressions. Moreover, the use of MRI-compatible video equipment, which is necessary for capturing facial expressions during scanning, is prohibitively expensive, adding another layer of difficulty to conducting such research.

Finally, owing to its relatively low temporal resolution, fMRI is not well suited for investigating rapidly changing aspects of facial expressions, such as micro-expressions—unconscious facial movements characterized by extremely short durations and small amplitudes [95,96]. However, beyond fMRI, several neuroimaging tools offer varying levels of temporal and spatial resolution and do not impose the same constraints on capturing full facial expressions during experiments. Electroencephalography (EEG), for example, enables the measurement of event-related potentials (ERPs) using only an electrode cap, leaving most of the face unobstructed. This set-up permits relatively unrestricted, high-resolution video recording of facial expressions from multiple angles during experiments. However, EEG signals are particularly susceptible to contamination from facial muscle activity, posing a substantial risk of confounding effects and potentially leading to erroneous interpretations if not carefully controlled [97]. Meanwhile, magnetoencephalography (MEG) is a non-invasive technique for mapping human brain electrophysiology, providing whole-brain coverage with high spatio-temporal resolution [98,99]. Recent technological developments in MEG have led to the introduction of optically pumped magnetometers (OPMs), a new wearable MEG technology that offers several potential advantages, including enhanced signal strength, adaptability to head size and greater tolerance to movement [99–102]. These properties are particularly useful for recording neural responses during facial expressions. However, the millisecond temporal resolution advantage of EEG and MEG source imaging can be offset by their rapidly decreasing sensitivity to neural sources located deeper in the brain [103]. Another promising tool is functional near-infrared spectroscopy (fNIRS), which is particularly well suited for multimodal research [104]. Compared with fMRI and EEG, fNIRS is less sensitive to motion artefacts associated with participants’ physical movement and allows unobstructed observation of the face [105]. Notably, fNIRS enables the assessment of interpersonal neural synchronization through the simultaneous measurement of neural activity from multiple socially interacting individuals, a paradigm known as hyperscanning [106]. This makes fNIRS especially advantageous for studying facial expression production and perception alongside neural activity in interactive contexts. Given the central role of facial expressions in interpersonal emotional communication, fNIRS offers a promising avenue for more ecologically valid investigations of facial function. Nevertheless, it remains unclear whether strong facial muscle contractions may influence fNIRS signals through stretching or displacement of the scalp. A further complementary approach is transcranial magnetic stimulation (TMS). Repetitive TMS (rTMS) is a neuromodulation technique that can either excite or inhibit targeted cortical regions, thereby enabling causal manipulation of emotion-related neural states. For example, rTMS was used to inhibit motor and somato-sensory cortices innervating the cheek region to experimentally modulate smiling behaviour [107]. Such approaches facilitate the identification of brain regions involved in emotional processing and the functional role of facial expressions. However, a key limitation is that experimentally induced emotional states may differ substantially from naturalistic emotional experiences. To date, many TMS studies have focused on mood modulation in clinical populations, yet this line of research holds important potential for clarifying how facial expressions contribute to emotion regulation and affective experience. Although a variety of neurophysiological measures are available, each with complementary strengths and weaknesses in terms of temporal resolution, spatial resolution and invasiveness, fMRI-based approaches are particularly well suited for modelling large-scale brain networks and characterizing interactions across distributed regions over time. In this respect, fMRI provides an important systems-level framework for investigating the neural architecture of expression production. Continued methodological development in fMRI-based studies of facial expression is, therefore, warranted, while remaining attentive to the complementary contributions of other neuroscientific tools.

### (c) Addressing the gap

Although these limitations are challenging to overcome, potential strategies may help mitigate them. One useful parallel can be drawn from pain research in neuroscience, where fMRI studies have successfully employed heat stimulation as a reliable method for inducing and controlling subjective experiences. For example, Becerra and colleagues used carefully calibrated heat stimuli—41°C for non-painful and 46°C for painful sensations—to distinguish between varying levels of subjective experience [108]. Such approaches allow researchers to either apply uniform stimulation across participants or adjust the intensity relative to each individual's pain threshold [109,110]. As a result, pain research has often benefited from more tightly controlled experimental conditions than many other emotional domains of emotion research. Indeed, in two previous studies investigating the production of painful facial expressions, pain was elicited through heat stimuli tailored to each participant's pain threshold [83,88]. By extension, advancing the study of emotion will require methods that can not only reliably elicit affective states but also systematically manipulate their intensity and quality.

When investigating facial expressions that arise from emotional experiences, it is crucial to ensure that the induced emotions are sufficiently robust to produce meaningful and analysable expressions. One widely used method is autobiographical memory recall, in which participants are instructed to remember highly emotional events, such as their saddest or happiest experiences. This approach elicits emotions in a personalized manner, analogous to the way that individualized heat stimulation can be tailored to each participant in pain research. Importantly, the intensity of an emotional response is often linked to the vividness of the recalled autobiographical memory [111], making this method effective for generating strong affective states. However, unlike heat stimulation, autobiographical recall is less standardized and lacks an objective unit of measurement. The duration of recall varies across participants, and the emotional impact can depend on factors such as the recency and personal significance of the memory. Moreover, additional validation procedures are often required to confirm that the emotions elicited through memory correspond to the specific target emotion intended by the researcher.

Therefore, for more controlled experimental designs, relying on external stimuli is often more desirable. In recent years, naturalistic paradigms have gained traction in neuroscience as a means of eliciting more ecologically valid, real-life emotional experiences [112,113]. Such paradigms are engaging and relatable, allowing participants to remain attentive even within the constrained environment of an fMRI laboratory setting [113]. Importantly, independent validation studies can confirm whether the naturalistic stimuli reliably elicit the targeted emotions and assess the intensity of the induced responses, thereby improving experimental control. Nonetheless, several challenges remain. First, individual differences in emotional responses cannot be fully controlled; what evokes strong emotions in one person may elicit weaker responses in another. Second, prior exposure to a stimulus may attenuate its emotional impact. Third, perceived and experienced emotions in the naturalistic setting are not always aligned, underscoring the need to differentiate between them [114]. Given that our ultimate goal is to examine how experienced emotions manifest in facial expressions, it is essential to include post-experiment assessments that verify the type and intensity of emotions actually experienced during viewing.

As noted earlier, when a participant's face is partially obscured or distorted during scanning, one potential workaround is to have them rewatch the stimuli outside the scanner. However, this approach is not recommended because the emotional responses elicited during a rewatch are likely to differ from those experienced initially, thereby increasing the disconnect between brain and behaviour. Whenever possible, simultaneous recording of facial expressions and brain activity during scanning is preferable to ensure greater ecological and methodological validity.

In addition to FACS-based approaches, facial electromyography (EMG) offers an alternative method for analysing facial expressions. Facial EMG measures the electrical activity generated by muscle contractions, which can be analysed using the FACS coding framework by focusing on corresponding specific AUs [115]. Recordings can be obtained using either needle electrodes or surface electrodes, with the latter being more commonly used in psychological research owing to their non-invasive nature. Surface EMG captures signals from broader regions compared with the more localized activity recorded with needle electrodes [116]. Notably, one study combined simultaneous facial EMG and fMRI to investigate facial mimicry and its neural underpinnings during emotional expression observation [117]. Unlike video-based approaches, facial EMG does not require full-face recordings because it directly measures muscle activity through electrodes. A further advantage is its selectivity: only a subset of facial muscles needs to be monitored, and prior studies have predominantly focused on the corrugator and zygomaticus muscles [116,118]. However, facial EMG is not without limitations. It is highly sensitive to movement artefacts and attaching electrodes to the face can be unnatural [115], potentially introducing noise and interfering with spontaneous facial expressions. Nonetheless, given that fMRI itself requires participants to minimize motion, the concurrent use of facial EMG and fMRI may not introduce substantial additional drawbacks and could, in fact, be a practical methodological choice. Importantly, certain considerations should be taken into account when analysing facial expression measures. Facial expressions vary across individuals; some people naturally display broader, more frequent expressions, while others exhibit more subtle changes. To account for these individual differences, it is advisable to establish a baseline of each participant's facial activity and normalize subsequent expression measurements relative to this baseline before analysis.

## 4. Diversity in emotion research

One of the central controversies in affect science concerns the cultural universality of emotions. Research continues to yield conflicting findings regarding whether basic emotions and their expressions are truly universal or culturally constructed. Films are often employed as naturalistic stimuli in emotion research. However, their effectiveness is limited by cultural and linguistic specificity. Certain scenes may require historical or cultural knowledge for accurate interpretation, while wordplay and subtle nuances are accessible only to those familiar with the language. Therefore, using video as an emotional stimulus necessitates careful validation—ideally through cross-cultural validation studies or at minimum within the target population. Rather than relying exclusively on stimuli from previous studies, researchers are encouraged to validate or develop appropriate materials for their own samples. As a notable example, a review of naturalistic stimuli in affective neuroimaging highlights that the majority of video clip stimuli used in research originate from Western countries (see table 1 in [114]). Similarly, large-scale datasets, such as the Healthy Brain Network (HBN) and the Human Connectome Project (HCP), also employ movie stimuli derived from Western cultures [119,120]. Asian-developed stimuli are seldom used in research and are normally used for only Asian samples (see [87] in table 1). Expanding the diversity of emotional stimuli across cultural contexts is, therefore, critical for advancing a more comprehensive and globally relevant science of emotion.

## 5. Towards a neuroscience of emotion production

Emotions are not only experienced internally but also expressed outwardly as social signals, with the face serving as one of the primary channels of expression. However, facial expressions are essential not only for perceiving emotions but also for conveying them. Consequently, examining how facial expressions are generated during subjective emotional experiences is of critical importance. Nevertheless, our review of the extant literature identified only 49 studies on the production of facial expressions, of which merely 10 examined expression during genuine emotional experiences (figure 3). This scarcity probably reflects both limited scholarly attention to the topic and the considerable methodological challenges it poses. The significance of facial expression research extends far beyond perception alone. A more balanced investigation of the three core domains of facial expressions—perception, experience and production—would substantially broaden our understanding of emotion.

Given that emotions inherently contain substantial social components, several considerations arise when studying the production of facial expressions. First, the facial expressions that accompany emotional experiences may differ depending on whether an individual is alone or in the presence of others. This leads to a second, closely related point: it is crucial to distinguish facial expressions that arise spontaneously from emotional experience from those that are intentionally produced for social communication. Because humans are fundamentally social beings, they often regulate their facial expressions during interpersonal interactions. Individuals may find themselves unable—or unwilling—to display their genuine emotional states. They may exaggerate or attenuate those states or even express emotions that they are not truly experiencing. As such, expressions produced in solitude may diverge substantially from those produced in social contexts, and these differences may further vary depending on who the interaction partner is. According to previous research using EEG [84], the authors expected that inner emotional–motivational processes (putatively indexed by frontal EEG asymmetry) could be dissociated from emotional facial expressions, given the influence of display rules. If there such display rules truly exist, operating at the conscious or unconscious level, research on facial expression production must remain attentive to the fact that emotional experience and emotional expression do not always align. Importantly, this discrepancy should not be viewed solely as a methodological limitation. Rather, it presents a valuable opportunity to uncover the mechanisms linking emotional experience and expression. Experimental manipulations—such as comparing solitary conditions with interactions involving unfamiliar individuals—can illuminate how social context shapes the relationship between experience and expression. Addressing the distinction between facial movements linked to emotional experience and those serving communicative or social signalling functions may require specifically designed experimental paradigms. For example, researchers could manipulate contextual expectations by informing participants that their facial expressions will be recorded and later evaluated by others to infer their emotional state and compare this with a condition in which no such evaluation is anticipated. Similarly, comparing facial expressions during the same task performed alone versus with other people (e.g. strangers or friends) may help clarify how social context influences expression production. Such designs may help determine whether emotional experience occurs first and is subsequently modulated before expression, whether modulation already emerges during the experience itself or whether only the expression stage is subject to social regulation. Against this backdrop, it becomes essential for research on facial expression production to be explicit about the mechanisms to be studied—namely, whether facial expressions are treated as automatic read-outs of internal emotional states, or socially learned and strategically regulated signals, or the outcome of a complex interplay between them.

Moreover, considering the process by which emotional experiences are elicited by antecedent events and translated into facial expressions suggests further several considerations. First, emotions are flexible and context-sensitive; thus, the relationship between stimuli and expressions may not be one-to-one. Multiple types of antecedent events may give rise to similar facial expressions, indicating that research designs should account for variability in eliciting conditions. Naturalistic paradigms with greater ecological validity may offer a promising avenue for capturing this variability. These also underscore the importance of taking socio-cultural context into account in research on expression production. Display rules may vary across cultures, and previously observed cultural differences in perception of facial expression may, in part, stem from differences in expression production.

Furthermore, emotional experiences are typically accompanied by internal physiological responses. Unfortunately, the precise relationship between physiological responses and facial expressions remains to be fully clarified. Given that physiological activity constitutes an important component of emotion, collecting physiological data alongside facial expression measures may help elucidate how internal responses relate to observable expressive behaviour and contribute to a more comprehensive understanding of expression production.

Taken together, the social nature of emotion underscores that emotional facial expressions extend well beyond the prototypical configurations associated with Ekman's basic emotions. In real-world social contexts, a neutral face can serve as an emotional signal, a polite smile may mask awkwardness, and expressions of anger may be intentionally suppressed to maintain social harmony. Thus, emotional expressions often reflect not only internal emotional states but also the social goals and demands of the situation, highlighting the need for theoretical and empirical approaches that account for the dynamic and context-dependent nature of expression production. We believe that alongside existing behavioural approaches, further systematic research on the production of facial expressions from a neuroscientific perspective that incorporates these considerations will move the field one step closer to a universal definition of emotion.

## Supplementary Material



## Data Availability

Supplementary material is available online [121].

## Theme

One contribution of 19 to a theme issue ‘Mechanisms, development, phylogeny and functions of emotional expressions’.
