# Supplementary material for: From seeing to showing: towards a neuroscience of emotion production
Source: Philos Trans R Soc Lond B Biol Sci. 2026 Sep 24;381(1959):20250136. doi: 10.1098/rstb.2025.0136 (PMC13644780; doi:10.1098/rstb.2025.0136)
Supplement: Supplementary file 1 [file rstb.2025.0136.zip › rstb.2025.0136/rstb20250136_si_001.pdf]

From seeing to showing: Toward a neuroscience of emotion production

Seohyeon Lee[1], Solha Hwang[1], & M. Justin Kim[1,2,3]

[1]Department of Psychology, Sungkyunkwan University, Seoul, South Korea
[2]Department of Brain Science and Engineering, Sungkyunkwan University, Suwon, South Korea
[3]Center for Neuroscience Imaging Research, Institute for Basic Science, Suwon, South Korea

List of Reviewed Articles

This provides a list of the 49 studies on facial expression production that were reviewed in the main text. Studies are listed in alphabetical order by the first author's last name.

Experience

1. Fox NA, Davidson RJ. 1986 Taste-elicited changes in facial signs of emotion and the asymmetry of brain electrical activity in human newborns. *Neuropsychologia.* **24**, 417–22. (doi:10.1016/0028-3932(86)90028-x)
2. Goldin PR, McRae K, Ramel W, Gross JJ. 2008 The neural bases of emotion regulation: reappraisal and suppression of negative emotion. *Biol. Psychiatry* **63**, 577–586. (doi:10.1016/j.biopsych.2007.05.031)
3. Grabell AS, Huppert TJ, Fishburn FA, Li Y, Jones HM, Wilett AE, Bemis LM, Perlman SB. 2018 Using facial muscular movements to understand young children's emotion regulation and concurrent neural activation. *Dev Sci.* **21**, e12628. (doi:10.1111/desc.12628)
4. Kunz M, Chen JI, Lautenbacher S, Rainville P. 2023 Brain mechanisms associated with facial encoding of affective states. *Cogn Affect Behav Neurosci.* **23**, 1281–1290. (doi:10.3758/s13415-023-01114-3)

5. Kunz M, Chen JI, Lautenbacher S, Vachon-Presseau E, Rainville P. 2011 Cerebral regulation of facial expressions of pain. *J Neurosci.* **31**, 8730–8738. (doi:10.1523/JNEUROSCI.0217-11.2011)
6. Mosley PE, van der Meer JN, Hamilton LHW, Fripp J, Parker S, Jeganathan J, Breakspear M, Parker R, Holland R, Mitchell BL et al. 2025 Markers of positive affect and brain state synchrony discriminate melancholic from non-melancholic depression using naturalistic stimuli. *Mol Psychiatry.* **30**, 848–860. (doi:10.1038/s41380-024-02699-y)
7. Ravaja N, Bente G, Kätsyri J, Salminen M, Takala T. 2018 Virtual character facial expressions influence human brain and facial EMG activity in a decision-making game. *IEEE Tran Affect Comput.* **9**, 285–298. (doi:10.1109/TAFFC.2016.2601101)
8. Zarei SA, Yahyavi SS, Salehi I, Kazemiha M, Kamali AM, Nami M. 2022 Toward reanimating the laughter-involved large-scale brain networks to alleviate affective symptoms. *Brain Behav.* **12**, e2640. (doi:10.1002/brb3.2640)
9. Zhao X, Liu Y, Chen T, Wang S, Chen J, Wang L, Liu G. 2022 Differences in brain activations between micro- and macro-expressions based on electroencephalography. *Front Neurosci.* **16**, 903448. (doi:10.3389/fnins.2022.903448)

Experience & Voluntary

1. Iwase M, Ouchi Y, Okada H, Yokoyama C, Nobezawa S, Yoshikawa E, Tsukada H, Takeda M, Yamashita, K, Takeda M et al. 2002 Neural substrates of human facial expression of pleasant emotion induced by comic films: a PET Study. *NeuroImage.* **17**, 758–768. (doi:10.1006/nimg.2002.1225)

Mimic

1. Achaibou A, Pourtois G, Schwartz S, Vuilleumier P. 2008 Simultaneous recording of EEG and facial muscle reactions during spontaneous emotional mimicry. *Neuropsychologia.* **46**, 1104–1113. (doi:10.1016/j.neuropsychologia.2007.10.019)
2. Benuzzi F, Ballotta D, Casadio C, Zanelli V, Porro CA, Nichelli PF, Lui F. 2023 "When You're Smiling": How Posed Facial Expressions Affect Visual Recognition of Emotions. *Brain Sci.* **13**, 668. (doi:10.3390/brainsci13040668)
3. Davis JD, Winkielman P, Coulson S. 2017 Sensorimotor simulation and emotion processing: Impairing facial action increases semantic retrieval demands. *Cogn Affect Behav Neurosci.* **17**, 652–664. (doi:10.3758/s13415-017-0503-2)
4. Godinez DA, Lumian DS, Crosby-Attipoe T, Bedacarratz AM, Zarolia P, McRae K. 2018 Overlapping and distinct neural correlates of imitating and opposing facial movements. *NeuroImage.* **166**, 239–246. (doi:10.1016/j.neuroimage.2017.10.023)
5. Gola KA, Shany-Ur T, Pressman P, Sulman I, Galeana E, Paulsen H, Nguyen L, Wu T, Adhimoolam B, Poorzand P et al. 2017 A neural network underlying intentional emotional facial expression in neurodegenerative disease. *NeuroImage Clin.* **14**, 672–678. (doi:10.1016/j.nicl.2017.01.016)
6. Han X, Luo S, Han S. 2016 Embodied neural responses to others' suffering. *Cogn Neurosci.* **7**, 114–127. (doi:10.1080/17588928.2015.1053440)
7. Horan WP, Iacoboni M, Cross KA, Korb A, Lee J, Nori P, Quintana J, Wynn JK, Green MF. 2014 Self-reported empathy and neural activity during action imitation and observation in schizophrenia. *NeuroImage Clin.* **5**, 100–108. (doi:10.1016/j.nicl.2014.06.006)
8. Iidaka T, Maesawa S, Kanayama N, Miyakoshi M, Ishizaki T, Saito R. 2024 Hemodynamic and electrophysiological responses of the human amygdala during face

imitation-a study using functional MRI and intracranial EEG. *Cereb Cortex*. **34**, bhad488. (doi:10.1093/cercor/bhad488)

9. Korb S, Malsert J, Rochas V, Rihs TA, Rieger SW, Schwab S, Niedenthal PM, Grandjean D. 2015 Gender differences in the neural network of facial mimicry of smiles--An rTMS study. *Cortex*. **70**, 101–114. (doi:10.1016/j.cortex.2015.06.025)
10. Krautheim JT, Dannlowski U, Steines M, Neziroğlu G, Acosta H, Sommer J, Straube B, Kircher T. 2019 Intergroup empathy: Enhanced neural resonance for ingroup facial emotion in a shared neural production-perception network. *NeuroImage*. **194**, 182–190. (doi:10.1016/j.neuroimage.2019.03.048)
11. Lee TW, Josephs O, Dolan RJ, Critchley HD. 2006 Imitating expressions: emotion-specific neural substrates in facial mimicry. *Soc Cogn Affect Neurosci*. **1**, 122–135. (doi:10.1093/scan/nsl012)
12. Lenzi D, Trentini C, Macaluso E, Graziano S, Speranza AM, Pantano P, Ammaniti M. 2016 Mothers with depressive symptoms display differential brain activations when empathizing with infant faces. *Psychiatry Res Neuroimaging*. **249**, 1–11. (doi:10.1016/j.pscychresns.2016.01.019)
13. Leslie KR, Johnson-Frey SH, Grafton ST. 2004 Functional imaging of face and hand imitation: towards a motor theory of empathy. *NeuroImage*. **21**, 601–607. (doi:10.1016/j.neuroimage.2003.09.038)
14. Miyata K, Koike T, Nakagawa E, Harada T, Sumiya M, Yamamoto T, Sadato N. 2021 Neural substrates for sharing intention in action during face-to-face imitation. *NeuroImage*. **233**, 117916. (doi:10.1016/j.neuroimage.2021.117916)
15. Pohl A, Anders S, Chen H, Patel HJ, Heller J, Reetz K, Mathiak K, Binkofski F. 2017 Impaired Emotional Mirroring in Parkinson's Disease-A Study on Brain Activation during Processing of Facial Expressions. *Front Neurol*. **8**, 682.

(doi:10.3389/fneur.2017.00682)

16. Rymarczyk K, Żurawski Ł, Jankowiak-Siuda K, Szatkowska I. 2018 Neural Correlates of Facial Mimicry: Simultaneous Measurements of EMG and BOLD Responses during Perception of Dynamic Compared to Static Facial Expressions. *Front Psychol.* **9**, 52. (doi:10.3389/fpsyg.2018.00052)
17. Rymarczyk K, Żurawski Ł, Jankowiak-Siuda K, Szatkowska I. 2019 Empathy in Facial Mimicry of Fear and Disgust: Simultaneous EMG-fMRI Recordings During Observation of Static and Dynamic Facial Expressions. *Front Psychol.* **10**, 701. (doi:10.3389/fpsyg.2019.00701)
18. Vrticka P, Simioni S, Fornari E, Schluep M, Vuilleumier P, Sander D. 2013 Neural substrates of social emotion regulation: a FMRI study on imitation and expressive suppression to dynamic facial signals. *Front Psychol.* **4**, 95. (doi:10.3389/fpsyg.2013.00095)
19. Wild B, Rodden FA, Rapp A, Erb M, Grodd W, Ruch W. 2006 Humor and smiling: cortical regions selective for cognitive, affective, and volitional components. *Neurology.* **66**, 887–893. (doi:10.1212/01.wnl.0000203123.68747.02)

Mimic & Voluntary

1. Budell L, Kunz M, Jackson PL, Rainville P. 2015 Mirroring pain in the brain: emotional expression versus motor imitation. *PloS one.* **10**, e0107526. (doi:10.1371/journal.pone.0107526)
2. Krivan SJ, Caltabiano N, Cottrell D, Thomas NA. 2020 I'll cry instead: Mu suppression responses to tearful facial expressions. *Neuropsychologia.* **143**, 107490. (doi:10.1016/j.neuropsychologia.2020.107490)

Stimulation

1. Caruana F, Avanzini P, Gozzo F, Francione S, Cardinale F, Rizzolatti G. 2015 Mirth and laughter elicited by electrical stimulation of the human anterior cingulate cortex. *Cortex.* **71**, 323–331. (doi:10.1016/j.cortex.2015.07.024)
2. Caruana F, Gozzo F, Pelliccia V, Cossu M, Avanzini P. 2016 Smile and laughter elicited by electrical stimulation of the frontal operculum. *Neuropsychologia.* **89**, 364–370. (doi:10.1016/j.neuropsychologia.2016.07.001)
3. Yamao Y, Matsumoto R, Kunieda T, Shibata S, Shimotake A, Kikuchi T, Satow T, Mikuni N, Fukuyama H, Ikeda A et al. 2015 Neural correlates of mirth and laughter: a direct electrical cortical stimulation study. *Cortex.* **66**, 134–140. (doi:10.1016/j.cortex.2014.11.008)

Voluntary

1. Baker J, Van der Donck S, Boets, B. Korb S. 2025 Increasing perceived happiness in neutral faces by posing a smile: An electroencephalography (EEG) frequency-tagging study. *Emot.* **25**, 1596–1604. (doi:10.1037/emo0001514)
2. Beringer M, Wacker J, Recio G. 2022 Deliberate control of facial expressions in a go/no-go task: An ERP study. *Acta Psychol.* **230**, 103773. (doi:10.1016/j.actpsy.2022.103773)
3. Chiew KS, Braver TS. 2011 Neural circuitry of emotional and cognitive conflict revealed through facial expressions. *PloS one.* **6**, e17635. (doi:10.1371/journal.pone.0017635)
4. Coan JA, Allen JJ, Harmon-Jones E. 2001 Voluntary facial expression and hemispheric asymmetry over the frontal cortex. *Psychophysiology.* **38**, 912–925. (doi:10.1111/1469-8986.3860912)
5. Korb S, Grandjean D, Scherer K. 2008 Motor commands of facial expressions: the bereitschaftspotential of posed smiles. *Brain Topogr.* **20**, 232–238. (doi:10.1007/s10548-

008-0049-2)

6. Krautheim JT, Steines M, Dannlowski U, Neziroğlu G, Acosta H, Sommer J, Straube B, Kircher T. 2020 Emotion specific neural activation for the production and perception of facial expressions. *Cortex.* **127**, 17–28. (doi:10.1016/j.cortex.2020.01.026)
7. Kroczek LOH, Mühlberger A. 2025 Neural mechanisms underlying the interactive exchange of facial emotional expressions. *Soc Cogn Affect Neurosci.* **20**, nsaf001. (doi: 10.1093/scan/nsaf001)
8. Price TF, Hortensius R, Harmon-Jones E. 2013 Neural and behavioral associations of manipulated determination facial expressions. *Biol. Psychol.* **94**, 221–227. (doi:10.1016/j.biopsycho.2013.06.001)
9. Sel A, Calvo-Merino B, Tuettenberg S, Forster B. 2015 When you smile, the world smiles at you: ERP evidence for self-expression effects on face processing. *Soc Cogn Affect Neurosci.* **10**, 1316–1322. (doi:10.1093/scan/nsv009)
10. Shangguan C, Wang X, Li X, Wang Y, Lu J, Li Z. 2019 Inhibition and Production of Anger Cost More: Evidence From an ERP Study on the Production and Switch of Voluntary Facial Emotional Expression. *Front Psychol.* **10**, 1276. (doi:10.3389/fpsyg.2019.01276)
11. Steines M, Krautheim JT, Neziroğlu G, Kircher T, Straube B. 2020 Conflicting group memberships modulate neural activation in an emotional production-perception network. *Cortex.* **126**, 153–172. (doi:10.1016/j.cortex.2019.12.020)
12. Stewart JL, Coan JA, Towers DN, Allen JJ. 2011 Frontal EEG asymmetry during emotional challenge differentiates individuals with and without lifetime major depressive disorder. *J Affect Disord.* **129**, 167–174. (doi:10.1016/j.jad.2010.08.029)
13. Takehara H, Ishihara S, Iwaki T. 2020 Comparison Between Facilitating and Suppressing Facial Emotional Expressions Using Frontal EEG Asymmetry. *Front Behav*

Neurosci. **14**, 554147. (doi:10.3389/fnbeh.2020.554147)

14. Volynets S, Smirnov D, Saarimäki H, Nummenmaa L. 2020 Statistical pattern recognition reveals shared neural signatures for displaying and recognizing specific facial expressions. *Soc Cogn Affect Neurosci.* **15**, 803–813. (doi:10.1093/scan/nsaa110)
15. Wild B, Erb M, Eyb M, Bartels M, Grodd W. 2003 Why are smiles contagious? An fMRI study of the interaction between perception of facial affect and facial movements. *Psychiatry Res.* **123**, 17–36. (doi:10.1016/s0925-4927(03)00006-4)

Detailed information of studies about production of facial expression

Supplementary Table 1 presents extended information on the 10 studies from the above list classified as *Experience* or *Experience & Voluntary*.

Supplementary Table 1. Methodological details and key findings of the included studies.

<table>
<tr><td rowspan="6">Fox & Davidson, 1986</td><th>N (sample)</th><th>Emotion elicitation</th><th>Expression type</th><th>Expression measure</th><th>Neural measure</th></tr>
<tr><td>• N = 16
• Healthy infants aged 2–3 days</td><td>Joy, Disgust</td><td>Spontaneous; facial expressions generated with emotional experience</td><td>Analyzed using the maximally discriminative facial coding system coding system (MAX)</td><td>EEG</td></tr>
<tr><th colspan="5">Experiment</th></tr>
<tr><td colspan="5">• Taste; sugar, citric acid, water.
• Sugar for positive affect, citric acid for negative affect, and water for neutral.
• Facial expressions during taste perception were recorded, and simultaneously EEG was measured.
• No social interaction (alone).</td></tr>
<tr><th colspan="5">Results</th></tr>
<tr><td colspan="5">• Newborn infants show differences in the asymmetry of brain electrical activity in response to different taste conditions.
• Sugar water was associated with increased left-hemispheric activation, whereas water was associated with reduced right-hemispheric activity, and no clear hemispheric differences were observed in response to citric acid.
• Facial data suggest that the initial introduction of the pipette into the newborn infant’s mouths elicited an aversive response.
• Although facial expressions during the citric acid condition indicated disgust expression, the corresponding emotion-related hemispheric activation patterns were not consistently evident in neural activity.</td></tr>
<tr><td rowspan="4">Iwase et al., 2002</td><th>N (sample)</th><th>Emotion elicitation</th><th>Expression type</th><th>Expression measure</th><th>Neural measure</th></tr>
<tr><td>• N = 12
• Healthy volunteers aged 28–45 years</td><td>Joy (specifically, laughter)</td><td>Spontaneous or Voluntary</td><td>Facial EMG; electrodes on bilateral major zygomatic muscles and orbicularis oculi muscles</td><td>PET</td></tr>
<tr><th colspan="5">Experiment</th></tr>
<tr><td colspan="5">• Film clips; 12 comic clips and 8 silent clips.
• Participants watched clips while PET was measured concurrently.</td></tr>
</table>

	• Spontaneous condition: Subjects were instructed to respond to the comic film clips at their will by laughing or smiling if the clips were pleasant.<br>• Voluntary condition: Participants watched emotionally neutral film clips without humorous content and were instructed to pull bilateral lip corners upward mimicking a laughing or smiling face.<br>• No social interaction (alone).				
	**Results**				
	• Spontaneous condition: The magnitude of laughter/smiling showed a positive correlation with activity in the supplementary motor area (SMA) and the left putamen, indicating that emotional expression is linked to affective–motor integrative circuits rather than primary motor cortex (M1).<br>• Voluntary condition: EMG activity correlated with activation in bilateral M1 (facial area) and the SMA, suggesting that intentional facial expressions primarily engage core motor control circuits centered on M1.<br>• The distinct neural substrates between facial expression of pleasant emotion and voluntary facial movement indicated that the SMA and left putamen are of importance for emotional facial expression.				
Goldin et al., 2008	**N (sample)**	**Emotion elicitation**	**Expression type**	**Expression measure**	**Neural measure**
	• N = 17<br>• Healthy female participants (age M = 22.7 years; SD = 3.5)	Disgust	Spontaneous (including reappraisal, suppression)	Manual FACS coding by human	fMRI
	**Experiment**				
	• Video clips; 10 nonaffective neutral nature scenes and 30 disgust-inducing scenes.<br>• 4 conditions (watch-neutral, watch-negative, reappraise-negative, suppress-negative trials).<br>• Reappraisal condition: thinking objectively to decrease emotional reactivity.<br>• Suppression condition: suppressing facial expressions to conceal subjective experiences.<br>• Facial expressions during video clip viewing were recorded, while fMRI scanning was performed concurrently.<br>• No social interaction (alone).				
	**Results**				

<table>
<tr><td></td><td colspan="5">• Spontaneous facial expressions were only used to compare neutral and negative stimuli. Reappraisal and suppression facial expressions were focused on.
• Reappraisal produced greater down-regulation of negative emotion experience, while suppression produced greater reduction in disgust facial behavior.
• Reappraisal increased activity in the prefrontal cortex (PFC) regions and decreased amygdala and insula responses.
• Suppression reduced negative emotion experience and behavior but sustained elevated responses in amygdala and insula.
• Greater PFC BOLD responses for reappraisal during the early period only and for suppression during the late period only.</td></tr>
<tr><td rowspan="6">Kunz et al., 2011</td><td>N (sample)</td><td>Emotion elicitation</td><td>Expression type</td><td>Expression measure</td><td>Neural measure</td></tr>
<tr><td>• N = 34
• Healthy volunteers aged 18–30 years (M = 23.4; SD = 2.5)</td><td>Pain</td><td>Spontaneous</td><td>Manual FACS coding by human</td><td>fMRI</td></tr>
<tr><td colspan="5">Experiment</td></tr>
<tr><td colspan="5">• Heat stimuli (painful and non-painful for control).
• Pain stimuli were delivered during fMRI scanning, and facial expressions were recorded throughout all scans.
• Two additional scans were conducted in which participants with less facial expressiveness were explicitly instructed to express pain using facial expressions.
• No social interaction (alone).</td></tr>
<tr><td colspan="5">Results</td></tr>
<tr><td colspan="5">• Painful heat stimuli elicited greater brow lowering, tightening of the muscles surrounding the eyes, and levator contraction compared with non-painful heat stimuli.
• Lower facial expressiveness was associated with increased activation in the prefrontal cortex and the caudate nucleus, indicating engagement of neural mechanisms underlying expressive suppression. The mPFC may serve as a key region implementing and representing display rules.
• Higher facial expressiveness was associated with increased activation in motor regions and pain-processing pathways. However, because activation in the M1 was not simply coupled with deactivation of the mPFC, facial expression generation and suppression appear to be independent processes.</td></tr>
</table>

<table>
<tr><td></td><td colspan="5">• Given that subjective pain ratings did not differ as a function of facial expressiveness, facial expressions capture aspects of pain processing not reflected in self-report measures.</td></tr>
<tr><td rowspan="6">Ravaja et al., 2018</td><td>N (sample)</td><td>Emotion elicitation</td><td>Expression type</td><td>Expression measure</td><td>Neural measure</td></tr>
<tr><td>• N = 28
• Undergraduates aged 20–35 years (M = 24.5)</td><td>Angry, happy, sad, pleasantly relaxed</td><td>Spontaneous</td><td>Facial EMG; electrodes on the left corrugator supercilii, zygomaticus major, and orbicularis oculi muscle regions</td><td>EEG</td></tr>
<tr><td colspan="5">Experiment</td></tr>
<tr><td colspan="5">• Interacting with a virtual character (VC) in the Iterated Prisoner's Dilemma game.
• Facial Expression of VC: angry, happy, sad, pleasantly relaxed, neutral, no-face, and responsive facial expressions dependent on the emotional state of the participant as assessed in real-time by facial EMG (congruent/incongruent).
• Facial expressions were recorded concurrently with EEG.
• Analyze the data separately for the pre-decision versus post-decision phases.
• Presence of social interaction; cooperate with or defect from a virtual character.</td></tr>
<tr><td colspan="5">Results</td></tr>
<tr><td colspan="5">• VC facial expressions elicited congruent facial muscle activity.
• Human facial expressions elicited by VC emotional expressions would depend on the previous interactional events and be affected by display rules.
• Angry VC expression would be expected to signal antagonism rather than willingness to engage in cooperative behavior.
• Pre-decision: the decrease in relative left frontal activation elicited by an angry VC was particularly pronounced when the participant had cooperated in the previous trial.
• Post-decision: when failing to achieve one's goal, people may sometimes express (by smiling) the opposite emotion of what they are actually feeling (potentially for the purposes of emotional coping).</td></tr>
<tr><td rowspan="2">Grabell et al., 2018</td><td>N (sample)</td><td>Emotion elicitation</td><td>Expression type</td><td>Expression measure</td><td>Neural measure</td></tr>
<tr><td>• N = 65</td><td>Frustration</td><td>Spontaneous</td><td>Manual FACS coding by human</td><td>fNIRS</td></tr>
</table>

	• Healthy young children aged 3–7 years (M = 5.04; SD = 1.3)				
	**Experiment**				
	• The Frustration Emotion Task for Children (FETCH); the game outcomes were manipulated to induce winning or losing.<br>• Facial expressions were recorded concurrently with fNIRS measurement.<br>• No social interaction (alone).				
	**Results**				
	• Children produced significantly more positive expressions than negative expressions during frustration to reflect a masking or display rules-related behavior to cope with or adapt to the stressor.<br>• Heightened LPFC response occurred in children who had infrequent negative expressions, suggesting the LPFC supports emotion regulation, as evidenced by control of facial display.				
Zarei et al., 2022	**N (sample)**	**Emotion elicitation**	**Expression type**	**Expression measure**	**Neural measure**
	• N = 25<br>• Healthy college students aged 19–30 years	Joy (specifically, laughter)	Spontaneous	Automated FACS coding using Face reader software	Quantitative EEG (qEEG)
	**Experiment**				
	• Video clips; 2 funny clips and 2 neutral clips.<br>• Facial expressions during viewing of the second funny clip and the second neutral clip were recorded, while qEEG was measured concurrently (An eye tracker was worn during viewing of the each first clip).<br>• No social interaction (alone).				
	**Results**				

<table>
<tr><td></td><td colspan="5">• Facial expressions during viewing of funny clips differed significantly from those during viewing of neutral clips.
• The heart rate variability, the trapezius muscle surface electromyography measures, the average pupil size and average fixation drifts were significantly different between funny and neutral clips, showing significant changes in the autonomic nervous system dynamics when laughing.
• There were significant differences in absolute power between the states where the participants watching the funny and neutral videos.
• The frontotemporal network (FTN) theta coherence z-score was increased when watching the funny clips.
• The current source density (CSD) for alpha band represented the highest gain in the right inferior frontal region when watching the funny clips.</td></tr>
<tr><td rowspan="6">Zhao et al., 2022</td><th>N (sample)</th><th>Emotion elicitation</th><th>Expression type</th><th>Expression measure</th><th>Neural measure</th></tr>
<tr><td>• N = 78
• Healthy participants aged 17–22 years</td><td>Amusement, Fear</td><td>Spontaneous but trying not to show facial expression</td><td>Manual FACS coding by human</td><td>EEG</td></tr>
<tr><th colspan="5">Experiment</th></tr>
<tr><td colspan="5">• Video clips; Chinese comedy film clips and variety shows for 3 amusement videos, classic scary films for 3 fear videos, and 1 neutral clip.
• Participants were instructed to maintain a neutral facial expression during video clip viewing, with performance-contingent rewards provided.
• Facial expressions during video viewing were recorded, while EEG was measured concurrently.
• No social interaction (alone).</td></tr>
<tr><th colspan="5">Results</th></tr>
<tr><td colspan="5">• Both micro- and macro-expressions recruited the left temporal and prefrontal lobes across emotional conditions, which may reflect the involvement of neural processes related to the inhibition of facial control.
• Under positive emotions, micro-expressions exhibited relatively stronger activation in the premotor cortex, supplementary motor cortex, and middle frontal gyrus regions, potentially suggesting that micro-expressions during positive emotion are more closely associated with facial control mechanisms.
• Under negative emotions, micro-expressions showed comparatively weaker activation in the somatosensory cortex and angular gyrus, which may indicate that micro-expressions in negative emotion are more closely related to the emotional intensity.</td></tr>
<tr><td rowspan="2">Kunz et al., 2023</td><th>N (sample)</th><th>Emotion elicitation</th><th>Expression type</th><th>Expression measure</th><th>Neural measure</th></tr>
<tr><td>• N = 27</td><td>Pain</td><td>Spontaneous</td><td>Manual FACS coding by human</td><td>fMRI</td></tr>
</table>

<table>
<tr><td rowspan="5"></td><td>• Healthy volunteers aged 18–30 years (M = 22.7; SD = 3.2)</td><td></td><td></td><td></td><td></td></tr>
<tr><td colspan="5">Experiment</td></tr>
<tr><td colspan="5">• Heat.
• Pain was induced using heat stimuli during fMRI scanning, while facial expressions were recorded concurrently.
• No social interaction (alone).</td></tr>
<tr><td colspan="5">Results</td></tr>
<tr><td colspan="5">• On average, visible facial expressions of pain occurred during only about 30% of the period of sustained pain stimulation. Facial expressions of pain showed substantial intra- and inter-individual variability.
• When pain was encoded in facial expressions, increased activation was observed in regions associated with facial motor activity as well as pain-processing regions, indicating enhanced pain-related neural activity at moments of facial expression.
• Encoding pain in facial expressions was accompanied by decreased prefrontal activation, suggesting that the prefrontal cortex regulates the degree to which emotional states are expressed facially. This supports the hypothesis that facial encoding of pain is generally suppressed by prefrontal systems and emerges when this regulatory control is released.</td></tr>
<tr><td rowspan="4">Mosley et al., 2025</td><td>N (sample)</td><td>Emotion elicitation</td><td>Expression type</td><td>Expression measure</td><td>Neural measure</td></tr>
<tr><td>• N = 70
• 30 melancholic; 40 non-melancholic participants (M = 44.0 years; SD = 12.8)</td><td>Positive affect</td><td>Spontaneous</td><td>Automated FACS coding using OpenFace</td><td>fMRI</td></tr>
<tr><td colspan="5">Experiment</td></tr>
<tr><td colspan="5">• 20-minute video clips; a routine by a stand-up comedian (‘Ricky Gervais: Animals’) and emotionally evocative movie (‘The Butterfly Circus’).
• Facial expressions were recorded while participants viewed Ricky Gervais: Animals (outside the scanner).</td></tr>
</table>

	• Participants viewed The Butterfly Circus during fMRI scan (inside the scanner).<br>• No social interaction (alone).
	**Results**
	• Facial expression and neural data were not collected simultaneously and were obtained during viewing of different stimuli.<br>• The melancholic group showed significantly reduced mean activity in six smile-related AUs (6, 7, 9, 12, 14, and 25).<br>• Hidden Markov Model analysis revealed key between-group differences in brain activity in response to positively valenced stimuli.<br>• Higher activity in AUs involved in positive expression was associated with lower activation in two cerebellar clusters; however, in melancholic patients, the correlation between cerebellar activity and AU activity was more blunted.
